# Retention and Risk Factors for Attrition in a Large Public Health ART Program in Myanmar: A Retrospective Cohort Analysis

**DOI:** 10.1371/journal.pone.0108615

**Published:** 2014-09-30

**Authors:** Aye Thida, Sai Thein Than Tun, Sai Ko Ko Zaw, Andrew A. Lover, Philippe Cavailler, Jennifer Chunn, Mar Mar Aye, Par Par, Kyaw Win Naing, Kaung Nyunt Zan, Myint Shwe, Thar Tun Kyaw, Zaw Htoon Waing, Philippe Clevenbergh

**Affiliations:** 1 The Union Office in Myanmar, International Union Against Tuberculosis and Lung Disease, Mandalay, Myanmar; 2 Infectious Diseases Programme, Saw Swee Hock School of Public Health, National University of Singapore, Singapore; 3 Innovation Unit, Médecins Sans Frontières, Geneva, Switzerland; 4 Maths and Statistics Help Centre, James Cook University, Singapore; 5 Medical Care Division, Department of Health, Mandalay, Myanmar; 6 National AIDS Program, Department of Health, Nay Pyi Taw, Myanmar; 7 Disease Control Division, Department of Health, Nay Pyi Taw, Myanmar; UNAIDS, Trinidad And Tobago

## Abstract

**Background:**

The outcomes from an antiretroviral treatment (ART) program within the public sector in Myanmar have not been reported. This study documents retention and the risk factors for attrition in a large ART public health program in Myanmar.

**Methods:**

A retrospective analysis of a cohort of adult patients enrolled in the Integrated HIV Care (IHC) Program between June 2005 and October 2011 and followed up until April 2012 is presented. The primary outcome was attrition (death or loss-follow up); a total of 10,223 patients were included in the 5-year cumulative survival analysis. Overall 5,718 patients were analyzed for the risk factors for attrition using both logistic regression and flexible parametric survival models.

**Result:**

The mean age was 36 years, 61% of patients were male, and the median follow up was 13.7 months. Overall 8,564 (84%) patients were retained in ART program: 750 (7%) were lost to follow-up and 909 (9%) died. During the 3 years follow-up, 1,542 attritions occurred over 17,524 person years at risk, giving an incidence density of 8.8% per year. The retention rates of participants at 12, 24, 36, 48 and 60 months were 86, 82, 80, 77 and 74% respectively. In multivariate analysis, being male, having high WHO staging, a low CD4 count, being anaemic or having low BMI at baseline were independent risk factors for attrition; tuberculosis (TB) treatment at ART initiation, a prior ART course before program enrollment and literacy were predictors for retention in the program.

**Conclusion:**

High retention rate of IHC program was documented within the public sector in Myanmar. Early diagnosis of HIV, nutritional support, proper investigation and treatment for patients with low CD4 counts and for those presenting with anaemia are crucial issues towards improvement of HIV program outcomes in resource-limited settings.

## Introduction

### HIV epidemiology and ART coverage in Myanmar

Myanmar has a concentrated HIV epidemic and the global HIV prevalence in adults has been estimated at 0.53% in a recent modelling study [1]. In the same study, the HIV prevalence in high risk groups has been estimated at 9.6, 7.8 and 21.9% in female sex workers (FSW), men who have sex with men (MSM) and injecting drug users (IDU), respectively [1].

There has been a very significant increase in the number of patients receiving ART worldwide, and by the end of 2011, it had reached 20 times the 2003 levels [2]. ART coverage in the Asia-Pacific region increased from 52% in 2009 to 56% in 2011 [3]. By the end of 2011, 40,128 people were receiving ART in Myanmar, which was double the number observed in 2009 [1]. By the end of 2012, 34,733 (65%) patients on ART were followed in the private sector (international NGOs) and 18,976 (35%) patients in the public sector. As per the Asia Epidemic Model (AEM) 2010–2015, the total numbers of people in need of ART in 2013, 2014 and 2015 are estimated at 125,043; 124,286 and 123,915 people respectively. With extended support from the Global Fund against AIDS, Tuberculosis and Malaria for a four-year period, 86% (106,000 patients) of the total affected population is slated to have access to ART by 2016 nationwide [personal communication, National AIDS Program]. To reach this important goal, the number of patients receiving treatment in the public sector need to be increased to 64,182 people, representing 61% of all patients on ART in Myanmar.

### Importance of retention in ART programs

As antiretroviral treatment becomes more readily available [2], the next major challenge for the National HIV Control program and the public sector will be to deliver high quality care [4]. In addition to viral load suppression which remains the key marker of treatment success, several other indicators have been used to measure the quality of HIV programs [5]–[7]. While retention in care of HIV-infected patients has been shown to reflect individual treatment success [8], [9], suboptimal retention is directly associated with negative outcomes including longer time to viral suppression [10], treatment failure and increased mortality [7], [8], [11], [12]. Factors associated with attrition (defined as the occurrence of death or loss to follow up) have been described previously [13]–[27]; the most common determinants include a late presentation to ART clinics, and a poor nutritional status at baseline.

### Previous studies in Myanmar

Determinants of treatment outcomes have been documented within the private sector in an ART cohort administered by Médecins Sans Frontières (MSF)-Holland in Myanmar; in this study the reported retention in care at 60 months reached 72% [95% CI: 70–74] [25]. However, neither the retention rates nor the determinants for attrition have been documented in the public sector. Therefore, the primary objectives in this study are to estimate the retention rates in a large ART program within the public sector in Myanmar, and to determine risk factors for attrition within this setting.

## Methods

### Ethics statement

Oral inform consent was obtained from all patients enrolled in the cohort; written informed consent was not collected as it was inadvisable to collect written documentation during the time of the military regime in Myanmar. The oral consent was obtained by marking a tick-box on the patient case form at enrollment after full discussion with a trained peer counselor, and kept in the patient's file. The protocol was approved by the Ethical Advisory Group (EAG) committee of the International Union Against Tuberculosis and Lung Disease (hereafter “The Union”) (EAG number 4/14). All data were treated confidentially and stored as anonymized data. Biological assays were performed as part of routine follow up and all tests results were anonymized.

### Setting

The Union has been implementing an Integrated HIV Care (IHC) program within the public sector in collaboration with the Department of Health, the National TB Program (NTP) and the National AIDS Program (NAP) since 2005. Initially, it was implemented at a single ART clinic in Mandalay. From that starting point, there was a steady expansion of the program so that by mid-2013, more than 15,000 patients were receiving ART in a total of 14 ART centers and 12 ART decentralized sites in 6 regions. The entry points for enrollment in this IHC program include TB clinics, pre-ART clinics run by the NAP, antenatal clinics, and hospitals located in the project area. The IHC program also accepts patients referred from other ART programs. There are no geographical limitations for enrollment in the IHC program (i.e. patients from the entire country have access to the program).

After enrolment, patients went through a standardized process including registration (allocation of a unique IHC code), baseline clinical assessment and laboratory testing, confirmation of eligibility by a selection committee and ART adherence counseling before ART initiation. Once patients are “stabilized”, generally 6 to 12 months after ART initiation at district or tertiary hospitals, they are transferred out to the nearest decentralization sites for chronic HIV care. Staff of the Ministry of Health provides clinical care for ART initiation and follow- up. The Union provides support including additional human resources (medical doctors, laboratory technicians …) comprehensive laboratory investigations (CD4, viral load testing since 2012, and other biochemical tests), drug procurement and supply management, data entry, and program monitoring. Self-management support from the People Living with HIV and AIDS (PLHA) network are deployed in all ART sites and were actively involved in the triage and registration, adherence counseling, and defaulter tracing. They also play an important role in providing financial assistance and social support for patients in distress.

The IHC program follows the national guideline for ART initiation, and uses AZT/3TC or D4T/3TC plus an NNRTI as first-line regimen, and protease inhibitor based regimens for second line treatment. In 2013, the national guidelines were updated to WHO recommendations and adopted TDF/3TC (FTC) as the preferred reverse transcriptase inhibitors. TB/HIV collaborative programs have been well established, and there is a cross-referral system between TB clinics and ART clinics for patients diagnosed at either of these sites. The enrolled patients also get support for necessary drugs for treatment of opportunistic infections (OI) and for required investigations if they are admitted to the hospital.

### Study design and population

**Figure 1:**
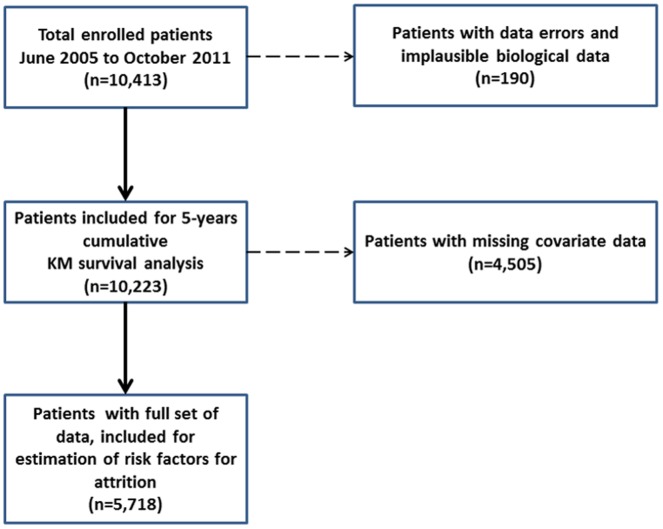
CONSORT diagram for analysis, with a total number of enrolled patients, number of patients for survival analysis and number of patients to estimate risk factors for attrition.

We retrospectively analyzed a total of 10,413 patients who were enrolled in our program between June 2005 and October 2011 and were followed up until April 2012 when censoring for this analysis occurred. After removing patients with data inconsistencies a total of 10,223 patients were considered for the 5-year cumulative survival analysis. For the logistic regression and survival analyses, patients with missing covariate data (primarily CD4 at initiation) were removed, leaving a final total of 5,718 patients for estimation of risk factors for attrition (Figure 1). The demographics of the full population and those with missing data are compared in table S1 in File S1. The profile of both groups is broadly similar; those with missing information were however less likely to receive a TB treatment at the time of ART initiation, but they were also more frequently reported with a previous ART history. This difference is largely due to the fact that the program absorbed an existing cohort of non-TB HIV-infected patients on ART, and that CD4 testing was only introduced in June 2006, while the program started one year earlier.

### Definitions for primary outcomes

The primary outcome of this study was attrition, which included death (all-cause) and loss to follow-up (LTF). Loss to follow-up was defined as patients who did not return to clinic within three months from their last scheduled appointment and who could not be traced by the PLHA network. A system was in place to extract the patient cohort code if they failed to come to the clinic and to allow the PLHA network to trace the patients back within two weeks. A small number of patients have been classified as LTF after referral to non-IHC ART clinics, as we are unable to investigate the continuum of care for these patients. The primary survival outcome was defined as those patients who were both alive and still in active follow-up at the end of April 2012. Survival time was measured from date of enrollment to date of death, last recorded clinical visit, or study censoring date.

### Data recording and storage

Routine patient medical data are recorded on paper forms by attending clinicians and Union staff. These forms are computerized in a password-protected database. All patient information was entered into the database using a unique coding system and the database is accessible only to data team members. We will make freely available any materials and data described in this publication that may be reasonably requested for the purpose of academic, non-commercial research.

### Statistical analysis

In this analysis, the models for attrition utilized recommendations for low-income settings which include CD4 count at baseline, sex, age, ART regimen (NNRTI vs. PI-based) and clinical stage (WHO stage I/II vs. III/IV) as independent predictors [28], [29]. Both multivariate logistic regression and survival models were utilized to assess risk factors for attrition.

We included in the multivariate models all variables associated with attrition from the univariate analysis (at p<0.20 level). Age was grouped as 18–30, 31–40, 41–50 and 51–60+ years; baseline CD4 cell count was dichotomized using the standard clinical cut-off of 100 cells per mm^3^ for HIV to AIDS progression [14], [15], [18], [20], [25]. Both the Akaike and Bayesian Information Criteria (AIC/BIC) were used to assess model parsimony. Confounding was also assessed: case-control tabulation and bivariate regression analyses were used to examine potential interactions (defined as 15% change in coefficient of the referent variable), and residual plots were examined for all multivariate models.

For both analyses the predictors examined were: patient characteristics (age, literacy, sex and alcohol consumption), and baseline clinical and laboratory markers (BMI, WHO staging, type of ART regimen, TB diagnosis and Cotrimoxazole prophylactic treatment, CD4 cell count, anaemia, elevated liver transaminase (ALT), prior OIs, and TB, hepatitis B and C serological status).

Proportion surviving was calculated using Kaplan-Meir methods; preliminary survival models used Cox proportional hazard methods. Due to extensive proportional hazard violations in multiple variables (anaemia, low BMI, and CD4<100 at enrollment), Royston-Parmer flexible parametric survival models were used [30]. These alternatives to standard Cox models add in splines to directly model the underlying hazard functions, and allow more comprehensive modeling and predictions.

Data management, exploratory data analyses and logistic regressions models were fit using the software R 2.15; survival analyses were performed using Stata 12.1, (College Station, Texas); all tests were two-tailed with α = 0.95.

### Management of missing variables

A comprehensive sensitivity analysis of the missing variables was performed and complete case analysis was performed for estimation of risk factors for attrition. The characteristics of the omitted and complete cases were similar with the exception of two variables- those with missing data were more likely to have a history of ART and less likely to have tuberculosis at baseline. As such, we are unable to assume the data are missing completely at random, which may introduce some bias in the estimates.

Our sensitivity analyses had two components. The first used simulated data, whereby data were randomly generated for missing values to assess the potential impact of ‘worst-case’ scenarios. The simulated data for each missing variable was assigned to one category and then used to compute adjusted odds ratios; this process was then repeated, and re-run with the opposite (for example, if the first set simulated a ‘male’ for all missing values, then the second set this was reversed, with all missing data coded as a ‘female’). The resulting sets of adjusted odds ratios were then compared.

To complement this analysis, the univariate odds ratios for the primary outcome were also compared between full case data and those with missing data (table S2 in File S1).

## Results

### Baseline Characteristics

The mean (±SD) for age was 36 (±8) years, and 61% of patients were male among a total of 10,413 patients. The demographic characteristics of study population at baseline such as age, sex, literacy, use of alcohol and HIV risk factors are shown in table 1. Clinical baseline characteristics (prior history of ART, WHO clinical Stage at ART initiation, CD4 at baseline, haemoglobin at baseline, HBV and HCV Status at baseline, BMI (baseline), type of ART regimen at baseline, Cotrimoxazole preventive therapy at baseline, tuberculosis treatment at ART initiation) are summarized in tables 2 and 3. Twenty four percent of patients had a prior ART history, 74% were classified as WHO stage III or IV and 46% had CD4 less than 100 cells/µl at ART initiation.

**Table 1:** Demographic baseline characteristics of study population.

		Alive and still in follow-up (N = 8,615)		Negative outcomes: death, lost to follow up (N = 1,798)		Total (N = 10,413)	
		n	%	n	%	n	%
Age	Mean (SD)	35.95 (7.98)		36.05 (8.52)		35.97 (8.08)	
	*Missing*					*26*	
Sex	Male	5090	59.1	1243	69.2	6333	60.9
	*Missing*					*10*	
Alcoholism	Habitual	674	8.7	177	11.0	851	9.1
	Social	1341	17.3	314	19.5	1655	17.7
	Never	5729	74.0	1120	69.5	6849	73.2
	*Missing*					*1058*	
Literacy	Not literate	798	9.5	210	11.9	1008	9.9
	Literate	7603	90.5	1553	88.1	9156	90.1
	*Missing*					*249*	
HIV risk factors	Heterosexual	7232	85.8	1510	85.6	8742	85.8
	MSM	141	1.7	32	1.8	173	1.7
	Sex Worker	16	0.2	3	0.2	19	0.2
	IDU	291	3.5	72	4.1	363	3.6
	Blood transfusion	305	3.6	51	2.9	356	3.5
	Mother to child	3	0.0	0	0.0	3	0.0
	Unknown	436	5.2	95	5.4	531	5.2
	*Missing*					*226*	

SD: Standard deviation; MSM: Men who have sex with men; IDU: Injecting drug user.

**Table 2:** Clinical baseline characteristics of study population.

		Alive and still in follow-up (N = 8,615)		Negative outcomes: death, lost to follow up (N = 1,798)		Total (N = 10,413)	
		n	%	n	%	N	%
Prior history of ART	No	6450	74.9	1463	81.4	7913	76.0
	Yes	2165	25.1	335	18.6	2500	24.0
	*Missing*					*0*	
WHO Clinical Stage at ART Initiation	Stage I/II	2452	28.6	223	12.5	2675	25.8
	Stage III/IV	6127	71.4	1568	87.5	7695	74.2
	*Missing*					*43*	
CD4 (cells/mm^3^) at baseline	Median [IQR]	117 (59–197)		74 (39–132)		109 (55–188)	
	0–100	3426	42.9	1012	62.6	4438	46.3
	>100	4551	57.1	604	37.4	5155	53.7
	*Missing*					*820*	
Haemoglobin at baseline	Normal	1778	22.8	162	9.9	1940	20.5
	Anaemia	6024	77.2	1477	90.1	7501	79.5
	Mean (SD)	11.0 (2.1)		10.0 (2.2)		10.8 (2.2)	
	*Not performed*					*972*	
HBV status at baseline	Negative	6552	90.9	1203	89.2	7755	90.7
	Positive	652	9.1	146	10.8	798	9.3
	*Not Performed*					*1860*	

IQR: Interquartile range.

WHO definition of Anaemia: Haemoglobin concentration <12 g/dL in women and <13 g/dL in men.

**Table 3:** Clinical baseline characteristics of study population, continued.

		Alive and still in follow-up (N = 8,615)		Negative outcomes: death, lost to follow up (N = 1,798)		Total (N = 10,413)	
		N	%	N	%	n	%
BMI (baseline)	Underweight (<18.5)	3426	44.7	1029	68.3	4455	48.6
	Normal	4241	55.3	478	31.7	4719	51.4
	Mean (SD)	19.3 (3.5)		17.4 (3.5)		19.0 (3.6)	
	*Missing*					*1239*	
HCV status at baseline	Negative	6814	94.9	1270	94.6	8084	94.8
	Positive	367	5.1	72	5.4	439	5.2
	*Not performed*					*1890*	
Type of ART regimen at baseline	NNRTI	8252	99.8	1686	99.4	9938	99.7
	PI	15	0.2	10	0.6	25	0.3
	*Missing*					*450*	
Cotrimoxazole preventive therapy at baseline	No	749	8.7	117	6.5	866	8.3
	Yes	7866	91.3	1681	93.5	9547	91.7
Tuberculosis treatment at ART initiation	No	5682	66.0	980	54.5	6662	64.0
	Yes	2933	34.0	818	45.5	3751	36.0
	*Missing*					*0*	

BMI: Body mass index; SD: Standard deviation; NNRTI: Non-nucleoside reverse transcriptase inhibitor; PI: Protease inhibitor.

### Retention Rate

**Figure 2:**
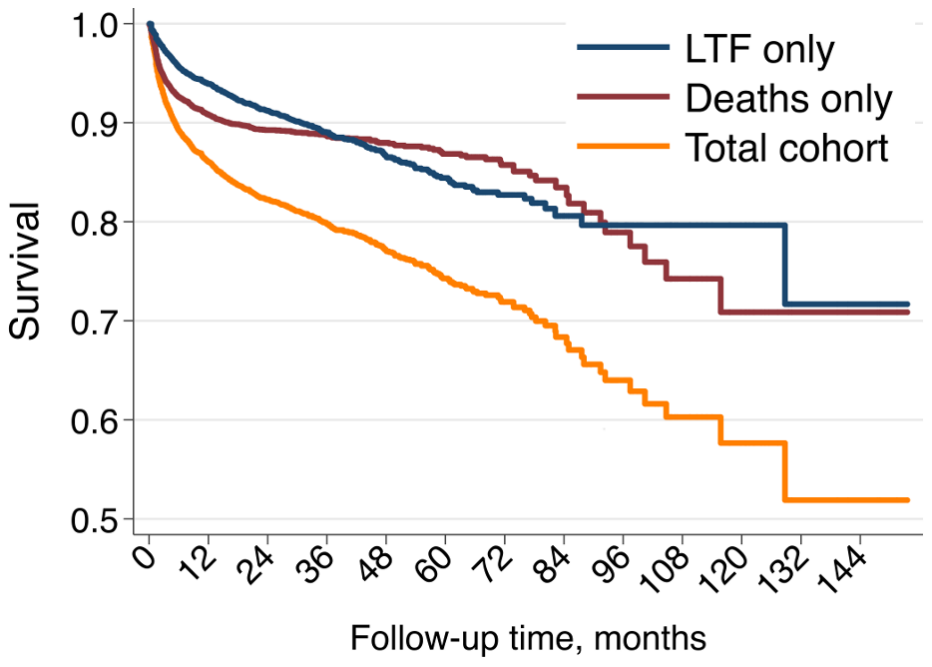
Unadjusted Kaplan-Meier survival curves, full cohort (N = 10,223).

The cumulative outcomes were death 909 (9%), loss to follow up 750 (7%) and in active follow up 8,564 (84%) for the cohort used for survival analysis (n = 10,223) with the median follow up duration of 13.7 months (interquartile range [IQR] 5.5–28.7). The corresponding retention rates and 95% CI of participants were 12 months (86; 85–87); 24 months (82; 81–83); 36 months (80; 79–81); 48 months (77; 76–78); 60 months (74; 73–76) (figure 2).

### Incidence density for attrition

Among 10,223 patients, 1,542 attrition [death or LTF] events occurred over 17,524 person-years at risk, with an annual incidence density of 8.8% (95% CI: 8.4–9.3) in our study. In comparison, 1,061 attrition [death or LTF] events occurred among 5,963 patients over 14,854 person years at risk with an incident density of 7.0% (95% CI: 6.7 to 7.6) per year in the private setting [25] (Table 4). However, we found that the cumulative attrition (death+ default) rate in the very first decentralized sites of IHC program was quite low (3%).

**Table 4:** Attrition Rate at 36-month follow up of the cohort in the public sector comparing with MSF (H) cohort.

Service Delivery Point	Participants (N)	Attrition Cases (N) [death+LTF] during 36 months of follow-up	Person years at risk (to 36 months)	Attrition Rate (%) Per Year	95% Confidence Interval
Private [MSF]	5,963	1,061	14,854	7%	(6.7–7.6) %
Public [The Union +DOH]	10,223	1,542	17,524	8.8%	(8.4–9.3) %

N: Number; LTF: Loss to follow up; MSF (H): Médecins Sans Frontières (Holland); DOH: Department of Health.

### Risk Factors for Attrition

Two complementary analyses were performed to investigate risk factors for attrition. The risk factors identified in logistic regression analysis are shown in Table 5 as odds ratios for primary outcome (death or loss-to-follow-up), and those identified in survival models are shown in Table 6 as hazard ratios from multivariate analysis.

**Table 5:** Prevalence percentages and odds ratios for primary outcome (death or loss-to-follow-up) from the logistic regression analysis.

			Bivariate Analysis	Multivariate Analysis	
	Alive and still in follow-up (N = 8615)	Negative outcomes: death, lost to follow up (N = 1798)	OR (95% C.I.)	OR (95% C.I).	p-value
Age: Mean (SD)	35.9 (8.0)	36.0 (8.5)	-	-	0.980
Sex: male	59.1	69.2	1.55 (1.39–1.73)	1.42 (1.21–1.65)	<0.001
Alcoholism: yes	26.0	30.5	1.25 (1.11–1.40)	0.98 (0.84–1.15)	0.804
Literate	90.5	88.1	0.78 (0.66–0.91)	0.68 (0.56–0.83)	<0.001
Prior history of ART	25.1	18.6	0.68 (0.60–0.78)	0.73 (0.58–0.90)	0.004
WHO Stage III/IV at baseline	71.4	87.5	2.81 (2.43–3.26)	2.36 (1.90–2.94)	<0.001
Underweight (BMI <18.5)	44.7	68.3	2.66 (2.37–3.00)	1.97 (1.71–2.26)	<0.001
CD4 at baseline <100	42.9	62.6	2.23 (1.99–2.48)	1.47 (1.29–1.69)	<0.001
Anaemia at baseline	77.2	90.1	2.69 (2.27–3.19)	1.67 (1.36–2.05)	<0.001
Tuberculosis treatment at baseline	34.0	45.5	1.62 (1.46–1.79)	0.90 (0.78–1.04)	0.140
PI base regimen	0.2	0.6	3.26 (1.46–7.28)	4.35 (1.22–15.49)	0.023
Cotrimoxazole preventive therapy at baseline	91.3	93.5	1.37 (1.12–1.67)	1.12 (0.84–1.50)	0.455

OR: Odds ratio; CI: Confidence interval; BMI: Body mass index; SD: Standard deviation; PI: Protease inhibitor; Anaemia: Haemoglobin concentration <12 g/dL in women and <13 g/dL in men.

**Table 6:** Flexible parametric survival model of potential determinants for attrition among study population (N = 5,718).

		Cox Univariate Analysis	Flexible Parametric Survival Model	
		HR, 95% C.I.	HR, 95% C.I.	p-value
Age	18–30	reference	reference	-
	31–40	0.96 (0.85–1.07)	0.90 (0.77–1.06)	0.201
	41–50	1.03 (0.89–1.20)	0.93 (0.76–1.13	0.466
	51–60 +	1.31 (1.06–1.61)	1.21 (0.90–1.64)	0.194
Enrollment group	Early	reference	reference	-
	Later	1.27 (1.14–1.42)	1.09 (0.94–1.26)	0.250
Sex: male		1.43 (1.29–1.59)	1.37 (1.17–1.61)	<0.001
Alcoholism: yes		1.26 (1.13–1.40)	1.05 (0.90–1.22)	0.542
Literate		0.83 (0.72–0.97)	0.81 (0.67–0.98)	0.031
Prior history of ART		0.52 (0.46–0.58)	0.61 (0.42–0.86)	0.005
Cotrimoxazole at baseline		1.34 (1.07–1.70)	1.58 (1.10–2.25)	0.011
WHO Stage III/IV at baseline		2.35 (2.00–2.76)	2.01 (1.58–2.57)	<0.001
Underweight (BMI<18.5) at baseline		2.55 (2.27–2.85)	2.06 (1.76–2.40)	<0.001
CD4 at baseline <100		2.73 (2.30–3.24)	1.74 (1.50–2.01)	<0.001
Anaemia at baseline		2.27 (1.88–2.75)	1.80 (1.41–2.30)	<0.001
Tuberculosis treatment at baseline		0.65 (0.59–0.72)	0.82 (0.71–0.94)	0.005
PI regimen at baseline		2.03 (1.01–4.07)	(ns #)	0.989

CI: Confidence interval; BMI: Body mass index; PI: Protease inhibitor; WHO definition of Anaemia: Haemoglobin concentration <12 g/dL in women and <13 g/dL in men.

#- not significant in univariate models.

Note: Early enrolment (1 Sep 2005 to 31 Dec 2009), and Late enrolment (1 Jan 2010 to 20 Oct 2011).

### Determinants for Attrition by Logistic Regression

After adjustment in a multivariate model, the significant factors identified were male sex [OR 1.4, 95% CI: 1.21–1.65]; illiteracy [OR 1.47, 95% CI: 1.21–1.79]; prior history of ART [OR 0.73, 95% CI: 0.58–0.90]; WHO stage III or IV at baseline [OR 2.36, 95% CI: 1.90–2.94]; BMI<18.5 kg/m^2^ [OR 1.97, 95% CI: 1.71–2.26]; CD4 less than 100 cells/µl at baseline [OR 1.47, 95% CI: 1.29–1.69]; anaemia at baseline [OR 1.67, 95% CI: 1.36–2.05] and protease inhibitor based regimen [OR 4.35, 95% CI: 1.22–15.49].

### Determinants for Attrition by Parametric Survival Model

In the adjusted multivariate flexible parametric model, the independent risk factors for attrition were: BMI<18.5 [HR 2.06, 95% CI: 1.76 to 2.40]; WHO stage III or IV at baseline [HR 2.01, 95% CI: 1.58 to 2.57]; anaemia at baseline [HR 1.80, 95% CI: 1.41 to 2.30], and baseline CD4 count <100 cells/µl [HR 1.74, 95% CI: 1.50 to 2.01].

Two factors showed marginally significant increased hazard: Cotrimoxazole treatment at baseline [HR 1.58, 95% CI: 1.10 to 2.25], and male gender [HR 1.37, 95% CI: 1.17 to 1.61].

**Figure 3:**
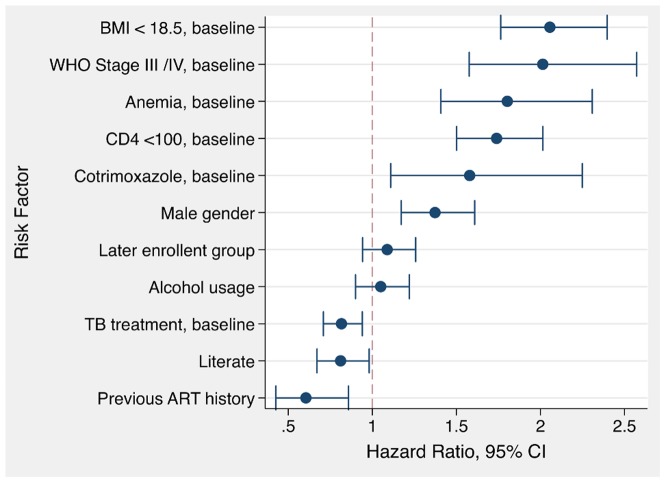
Adjusted hazard ratios from flexible parametric survival analysis (N = 5,718).

Several factors showed a protective effect: previous ART at baseline [HR 0.61, 95% CI: 0.42 to 0.86], literacy [HR 0.81, 95% CI: 0.67 to 0.98] and TB treatment at baseline [HR 0.82, 95% CI: 0.71 to 0.94] (figure 3).

### Analysis of missing data

The comparison of baseline demographics between full-data cases and those with missing data are presented in table S1 in File S1. All population characteristics are broadly similar with the exceptions of a more frequent ART history and a lower proportion of TB treatment at ART initiation in the group with missing data.

The comparison of the univariate odds ratios between cases with full data and those with missing values (table S2 in File S1), shows small differences in the point estimates between the two groups, but no changes in the substantive conclusions based upon the 95% CIs, with the exception of “PI-based regimen” which becomes not significant in the missing data group (2.39, 95% CI: 0.8 to 7.15) and “not literate” where it becomes not significant in the missing data group (1.15, 95% CI: 0.84 to 1.58).

Finally, the comparison of the multivariate odds ratios with the simulated data found only minor differences in point estimates and confidence intervals of the odds ratios, with the exception of the history of ART, where the odds ratio crosses one in the cases with simulated data (table S3 in File S1).

## Discussion

Several studies conducted in low and middle income countries have provided estimates for retention in HIV cohorts; however, most of these studies analyzed a limited number of patients and described only short- to medium-term retention.

### Retention in other Asian cohorts

Studies from Cambodia documented the outcomes of ART for short and medium term; the incidence of mortality was 9.1 per 100 person-years which is similar to international standards [31], the two year retention estimate was 85.5% both in a cohort of 416 patients treated in a Médecins Sans Frontières/Ministry of Health program [32], and in a larger cohort of the Sihanouk Hospital Center of HOPE, Phnom Penh [13]. The national program of Vietnam reported retention rates after 6, 12, 24, and 36 months as 88.4% [95% CI: 86.8–89.9], 84.0% [95% CI: 81.8–86.0], 78.8% [95% CI: 75.7–81.6], and 74.6% [95% CI: 69.6–79.0] [27]. In Thailand the survival rates among HIV-TB co-infected patients were 96.1%, 94% and 87.7% at 12, 24 and 36 months, respectively [33]. The retention at 12 month and 24 month of the Chinese National Free Antiretroviral Treatment Program was 92% and 84% [34] and the 12-month retention among eight Asia Pacific countries ranged from 65% to 88% [35].

The National AIDS Program in Myanmar reported a national retention rate of 87% at 12 months after ART initiation for those who received ART in 2010 [1]. The IHC program retention rate at 5 years was 74% [95% CI: 73–76], which is similar to the one reported by MSF Holland in its private sector cohort (72% [95% CI: 70–74]) [25]. However, there was a significant difference in the incidence density for attrition between the cohorts: 7.1% [95% CI: 6.7–7.6] per year in MSF versus 8.8% [95% CI: 8.4–9.3] per year in IHC program.

Possible explanations for these differences include the patient to provider ratio, and the corresponding time within the clinic, which can be better in a private setting with greater resources relative to the public sector. While it is extremely challenging to have reliable conclusions on trends in retention rates and to produce consistent nationwide data from many resource-limited countries [2], comparing and combining the results of the two largest cohorts in Myanmar may permit the national AIDS program to track outcomes of ART programs as national figures.

### Variations in program definitions

Currently, there is no consensus on how to best assess program retention [9]; although a study that analyzed 111 facilities worldwide suggested defining failure as more than 180 days from the last scheduled appointment [36]. Other methods that have been used include counting missed visits [37], appointment adherence [8] and gaps in care; and the number of gaps within 6, 9, or 12-month periods [9]. Various cohorts have reported various retention rates including 35% in Cameroon [24], 74.6% in Vietnam [27] and 82% in the MSF program in Myanmar [25] at 36 month follow up.

### Consideration of missing data

There is considerable debate as to the optimal strategy to handle missing data in cohort studies, and no method is optimal under all circumstances [38].

In this analysis we have focused on cases with full available data (complete case data), to maximize the comparability to studies in similar settings that have appeared in the literature. While this has the potential for introducing biases if the nature of missing data has an influence on the primary outcome, the multiple sets of complementary sensitivity analyses we have performed suggest these effects are likely small. Moreover, the demographics (age, sex, etc.) of the patients with missing data are remarkably similar to the included patients, with two exceptions. A higher proportion of patients with missing data have history of prior ART, while a lower proportion have TB treatment at ART initiation. The broad agreement of our results with prior studies suggests that any biases in our data analysis are likely minor.

### Attrition

Many factors have been linked to attrition in various settings, including the type of ART regimen at initiation [39], time to start of ART [16], [33], nutritional deficiency and anaemia [13], [18], [20], [22], [25], higher education [40] and strategic placing of ART service delivery points [24]. By focusing on modifiable factors in this cohort analysis, we aimed to provide an evidence base to inform policy makers on how to improve outcomes in ART programs implemented in Myanmar and SE Asia.

### Comparison with a private sector cohort in Myanmar [25]

Our findings are similar to the results of the MSF cohort analysis. In The MSF cohort, high WHO staging, low BMI, male sex and age were found to be independent risk factors for attrition. Our study did not find a strong impact of age, but also identified that a low CD4 count and anaemia were risk factors for attrition; conversely, TB treatment at ART initiation, ART history and literacy were found to be predictors for the retention in care. The observed differences are likely to be due to differences in program structure, and potentially to some differences between the study populations.

### CD4

One of the main risk factors for attrition is low CD4 count at enrollment; this is a common feature which has been found in many other cohort studies [13]–[23]. Our study found an adjusted hazard ratio for attrition of 1.74 (95% CI: 1.50–2.01), much higher in patients with low CD4 at ART initiation. These finding reinforce the crucial importance of early HIV diagnosis and timely initiation of ART in the broader perspective of increasing survival rates [17], [21].

However, pooled data from 13 research cohorts from five sub-Saharan African (Benin, Burkina Faso, Cameroon, Cote d'Ivoire and Senegal) and two Asian (Cambodia and Laos) countries reported a lower survival not only in those patients with low CD4 but also for patients with a high CD4 count [19], which is likely due to the presence of co-morbidities.

### BMI, WHO staging and anaemia

Several studies exploring HIV attrition found independent associations between attrition and low BMI [13], [18], [20], [25], WHO staging III/IV [16], [18], [20], [21], [25], and anaemia [13], [18], [20], [22]. We found similar associations in our cohort, and these factors are even stronger predictors of attrition than low CD4 count. Our findings support the prioritization of nutritional support and of appropriate management of anaemia as key interventions within the scope of the comprehensive care activities proposed in ART clinics in Myanmar. These results also suggest that insufficient nutrition is potentially an important risk factor for attrition in the study population. Further research is needed to more fully explore this association within Myanmar.

### Sex and Cotrimoxazole preventive therapy

Although the association is weak, male gender is an independent risk factor for attrition. This is in line with the results from other studies [13], [15], [23]–[25].

In contrast to a report from Thailand [26], we have found that cotrimoxazole prophylactic prescription is a risk factor for attrition by parametric survival models. This association is unsurprising as all patients with low CD4 or high WHO staging (both conditions known as strong predictors for attrition), are also treated with cotrimoxazole. This association, however, persisted in the multivariate analysis. This association could be an artifact; the lower range of the confidence interval [HR = 1.58 (95% CI: 1.10 to 2.25)], suggests only a marginal 10% increase in risk of event. This result was not found in logistic regression and may represent a spurious association.

### Comparison of first and second line regimens

A review of 18 cohorts in Europe and North America concluded that patients on protease inhibitor had higher probability of treatment interruption or modification; this review also highlighted that those patients receiving tenofovir/emtricitabine based regimens had lower probability of treatment substitutions compared to non-standard regimens [41]. In our cohort, the odds for attrition are higher for those patients receiving protease inhibitor based regimens [OR 4.35, (95% CI: 1.22 to 15.49)] when compared with patients on an NNRTI based regimen. While the number of patients on PI included in the analysis is limited, it is vital to investigate further whether this increased risk is due to delays in referral from non-IHC sites, or instead to the regimen itself in this particular setting.

**Figure 4:**
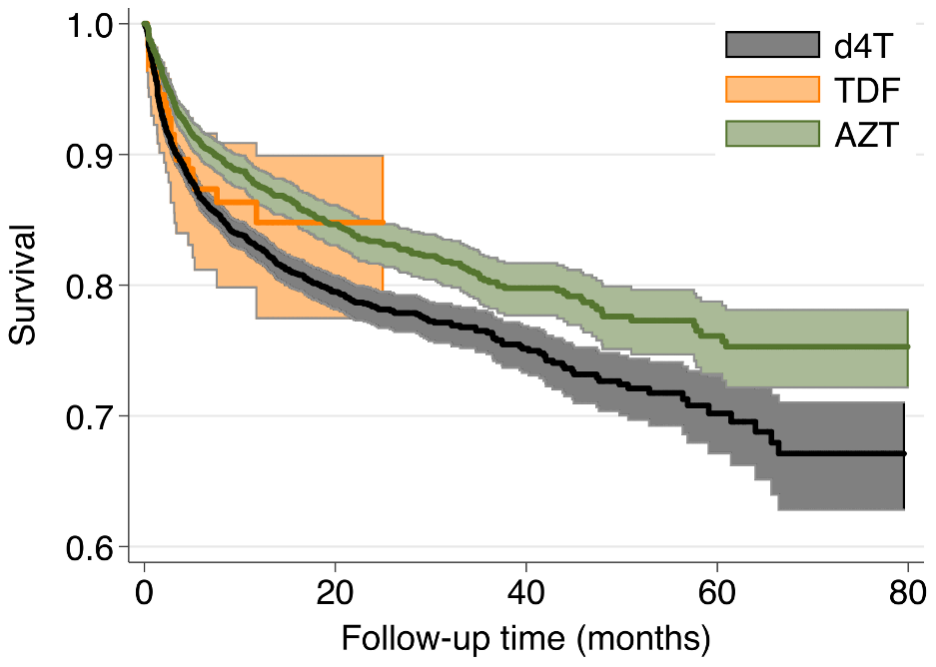
Unadjusted Kaplan-Meier curves, by first-line ART regimen.

Like other resource-limited settings, IHC programs in Myanmar used d4T as the main NRTI (67%) followed by AZT (31.2%) and TDF (2.3%) for ART initiation. A recent study from South Africa evaluated the effectiveness of TDF and reported that the adjusted hazard ratio for loss-from-care, when compared to TDF, was 1.5 (95% CI: 1.1–1.9) for d4T and 1.2 (95% CI: 1.1–1.4) for AZT [39]. In our study, only a small number of patients were treated with TDF, and a similar association was not found. However, the retention rate is higher in the unadjusted KM analysis for those on AZT when compared with patient receiving d4T-based regimens (p<0.0001), (Figure 4). As the effectiveness of modern cART regimens becomes widely documented it is worthwhile to recommend using those regimens whenever possible in ART programs [34]. In Myanmar, national antiretroviral treatment guidelines have been modified in mid-2013 to take into account the latest WHO recommendations and include the adoption of TDF/3TC as the preferred option for ART initiation.

### Attrition and TB co-infected patients, previous ART and Literacy

**Figure 5:**
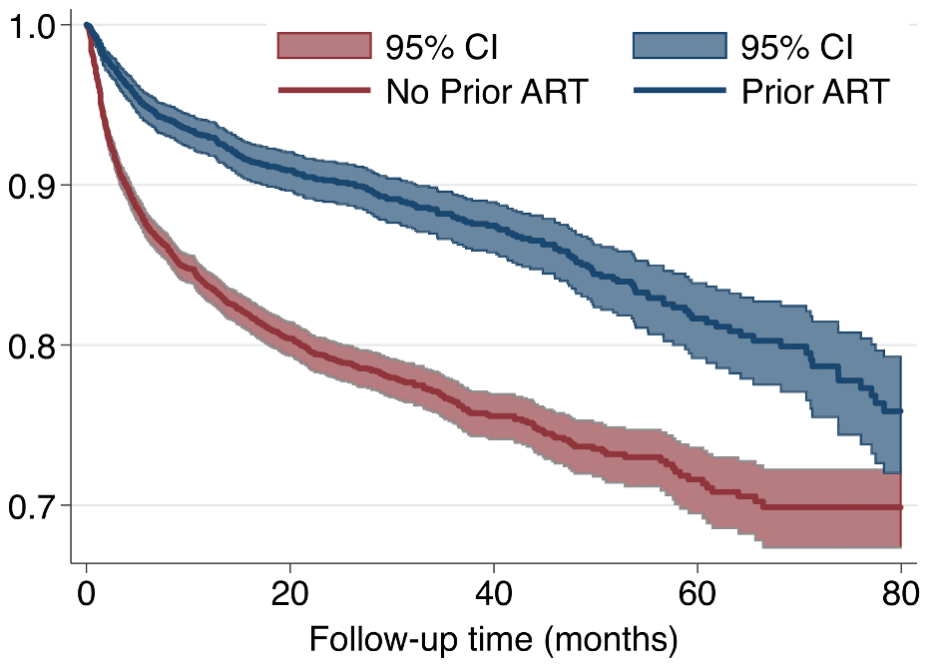
Unadjusted Kaplan-Meier survival curves of non-prior ART group and prior ART group, full cohort (N = 10,223). Log-rank test for equality of survival χ2 (1 df) = 122.10; p<0.001.

The protective factors for attrition include the administration of a TB treatment at ART initiation and a prior ART history (Figure 5); the better retention in care observed in these two groups can be explained by the more intensive counseling received by these patients in the TB clinics and by earlier ART care providers. In our cohort, 90% of study participants are literate and, not surprisingly, have a higher retention when compared to those with limited literacy. Increased educational attainment has been shown to have a strong positive impact on clinical outcomes [40] and more globally in cohort retention.

### Free of charge medical care, adherence support and defaulter tracing

A cluster randomized control trial in Vietnam reported that there was no significant difference in mortality rate between groups who received enhanced adherence versus a control groups without intervention [18]. A study from an ART clinic in Cameroon where patients had to pay for ART, found no difference in retention rates between before and after ART cost reduction [58%, 47% and 35% at 12, 24 and 36 months respectively] [24]. These findings highlight the importance of defaulter tracing in programs apart from the cost of ART. Implementation of support group activities in all project sites, peer involvement in defaulter tracing, and free access to all services are important aspects of the program in Myanmar that may explain these successes.

The modifiable risk factors for attrition we found could motivate attending clinicians to provide more comprehensive care to achieve both increased individual benefit and greater impact at the population level.

### ART site and decentralized sites

We found that the attrition remains quite low (3%) in the initial decentralized sites. Patients might have less constraint to come to these sites and better efforts might have been allocated to “stabilize” these patients (including the treatment of co-morbidities) before the transfer.

### Strength and limitations

One of the main strengths of this study is the sample size, which allowed us to investigate the retention in care and the risk factors for attrition in a large cohort of patients followed in the public sector in Myanmar. However, there are also some limitations. First, it should be acknowledged that a significant number of patients with missing data were excluded from the analysis, potentially introducing bias. Secondly, the study assessed a limited number of socio-demographic factors; we were not able to include in our analysis other important parameters such as the distance between the patients’ residence and the service delivery point [24] and the absorption capacity of the ART clinics [15]. Our study also mainly included patients who had already started ART while it would have also been important to try analyzing attrition before ART initiation [42]. We were also not able to analyze the biological markers after ART initiation [43]. Finally we were not able to capture any secular changes in standards of care and systematic changes within the health sector in Myanmar.

## Conclusion

Our study, conducted in a resource-limited setting, provides evidence about the quality and the effectiveness of a large ART program from the public sector in Myanmar. It also advocate for the feasibility of the long-term retention of a large cohort of patients on ART within an Integrated HIV Care program in Myanmar. Our result suggests that key improvements for future programs are early diagnosis of HIV and initiation of ART, comprehensive nutritional support to increase BMI to normal levels, and targeting those patients with low CD4 count and/or haemoglobin for intensive investigation and clinical management. Finally, a comprehensive study of patients in second-line therapy settings would be an important next step in addressing these programs gaps.

## Supporting Information

File S1 Contains Tables S1–S3. **Table S1**. Baseline demographics between cases with full data and those with missing predictors (N = 10,413). **Table S2**. Comparison of unadjusted OR for primary outcome between full data and missing case patients. **Table S3**. Comparison of full-case date and data with simulated outcomes to assess the potential impact of missing data.
(DOCX)
