# Supplementary material for: Retention and Risk Factors for Attrition in a Large Public Health ART Program in Myanmar: A Retrospective Cohort Analysis
Source: PLoS One. 2014 Sep 30;9(9):e108615. doi: 10.1371/journal.pone.0108615 (PMC4182661; doi:10.1371/journal.pone.0108615)
Supplement: File S1 — Contains Tables S1–S3. Table S1. Baseline demographics between cases with full data and those with missing predictors (N = 10,413). Table S2. Comparison of unadjusted OR for primary outcome between full data and missing case patients. Table S3. Comparison of full-case date and data with simulated outcomes to assess the potential impact of missing data. (DOCX) [file pone.0108615.s001.docx]

**Retention and risk factors for attrition in a large public health ART program in Myanmar: A retrospective cohort analysis**

|  | % missing | Complete data (%) | Incomplete data (%) |
| --- | --- | --- | --- |
| Age-Mean, (SD) | 0.3 | 36.0 (8.1) | 35.7 (8.3) |
| Is Male | 0.1 | 60.9 | 59.1 |
| Alcoholism (social/habitual) | 10.2 | 26.8 | 28.2 |
| Not Literate | 2.4 | 9.9 | 8.3 |
| History of prior ART | 0 | 24 | 44.3 |
| WHO Clin Stage at ART Initiation Stage III / IV | 0.4 | 74.2 | 71.2 |
| BMI (baseline) Underweight (<18.5) | 11.9 | 48.6 | 42.9 |
| CD4 at baseline < 100 | 7.9 | 46.3 | 44.5 |
| Anaemia at Baseline | 9.3 | 79.5 | 79.2 |
| TB Treatment at ART initiation | 0 | 36 | 25.4 |
| PI based regimen | 4.3 | 0.3 | 0.5 |
| Cotrimoxazole preventive therapy at baseline | 0 | 91.7 | 86.7 |
| Negative Outcome (death/loss to follow up) | 0 | 17.3 | 19.5 |

**Table S1.** Baseline demographics between cases with full data and those with missing predictors (N=10,413).

|  | All | | | | Incomplete Cases | | | |
| --- | --- | --- | --- | --- | --- | --- | --- | --- |
|  | Alive | Negative Outcome | OR | 95% CI | Alive | Negative Outcome | OR | 95% CI |
| Is Male | 59.1 | 69.2 | 1.55 | 1.39-1.73 | 57.2 | 67.4 | 1.55 | 1.29-1.86 |
| Alcoholism (social/habitual) | 26 | 30.5 | 1.25 | 1.11-1.4 | 26.8 | 33.6 | 1.38 | 1.1-1.73 |
| Not Literate | 9.5 | 11.9 | 1.29 | 1.1-1.51 | 8.1 | 9.2 | 1.15 | 0.84-1.58 |
| History of prior ART | 25.1 | 18.6 | 0.68 | 0.6-0.78 | 46.2 | 36.2 | 0.66 | 0.55-0.79 |
| WHO Clinical Stage at ART Initiation Stage III / IV | 71.4 | 87.5 | 2.81 | 2.43-3.26 | 68.3 | 83.3 | 2.32 | 1.85-2.91 |
| BMI (baseline) Underweight (<18.5) | 44.7 | 68.3 | 2.66 | 2.37-3 | 38.3 | 65.8 | 3.1 | 2.42-3.97 |
| CD4 at baseline <100 | 42.9 | 62.6 | 2.23 | 1.99-2.48 | 39.9 | 64.7 | 2.76 | 2.23-3.43 |
| Anaemia at Baseline | 77.2 | 90.1 | 2.69 | 2.27-3.19 | 75.9 | 91.8 | 3.56 | 2.51-5.05 |
| Tuberculosis Treatment at ART initiation | 34 | 45.5 | 1.62 | 1.46-1.79 | 22.5 | 37.3 | 2.05 | 1.7-2.47 |
| PI based regimen | 0.2 | 0.6 | 3.26 | 1.46-7.28 | 0.4 | 1 | 2.39 | 0.8-7.15 |
| Cotrimoxazole preventive therapy at baseline | 91.3 | 93.5 | 1.37 | 1.12-1.67 | 85.8 | 90.7 | 1.62 | 1.21-2.17 |

Table S 2. Comparison of unadjusted OR for primary outcome between full data and missing case patients.

|  | Complete Case Analysis | | Simulated data | | Simulated Data (reversed) | |
| --- | --- | --- | --- | --- | --- | --- |
|  | OR | 95% CI | OR | 95% CI | OR | 95% CI |
| Age | 1 | (1,1.01) | 1 | (0.99,1.01) | 1 | (0.99,1.01) |
| Is Male | 1.41 | (1.21,1.65) | 1.35 | (1.2,1.53) | 1.36 | (1.2,1.54) |
| Alcoholism (social/habitual) | 0.98 | (0.84,1.15) | 1.04 | (0.92,1.18) | 1.05 | (0.93,1.18) |
| Not Literate | 1.47 | (1.21,1.79) | 1.35 | (1.14,1.6) | 1.37 | (1.17,1.61) |
| History of prior ART | 0.72 | (0.58,0.9) | 0.9 | (0.79, 1.04) | 0.79 | (0.68, 0.93) |
| WHO Clinical Stage at ART Initiation Stage III / IV | 2.36 | (1.9, 2.94) | 1.94 | (1.65, 2.29) | 2.03 | (1.72, 2.39) |
| BMI (baseline) Underweight (<18.5) | 1.97 | (1.72,2.27) | 1.87 | (1.67,2.08) | 1.75 | (1.57,1.96) |
| CD4 at baseline < 100 | 1.47 | (1.29,1.69) | 1.48 | (1.33,1.66) | 1.63 | (1.46,1.82) |
| Anaemia at Baseline | 1.66 | (1.35, 2.05) | 1.81 | (1.53, 2.13) | 1.55 | (1.34, 1.79) |
| Tuberculosis Treatment at ART initiation | 0.9 | (0.78, 1.04) | 0.98 | (0.87, 1.1) | 0.98 | (0.87, 1.1) |
| PI based regimen | 4.35 | (1.22,15.46) | 4.54 | (2.04,10.07) | 2.62 | (2.03,3.38) |
| Cotrimoxazole preventive therapy at baseline | 1.12 | (0.83,1.49) | 1.18 | (0.96,1.45) | 1.2 | (0.97,1.49) |

Table S 3. Comparison of full-case date and data with simulated outcomes to assess the potential impact of missing data.
